# Dual-channel *P*-type ternary DNTT–graphene barristor

**DOI:** 10.1038/s41598-022-23669-w

**Published:** 2022-11-12

**Authors:** Yongsu Lee, Seung-Mo Kim, Kiyung Kim, So-Young Kim, Ho-In Lee, Heejin Kwon, Hae-Won Lee, Chaeeun Kim, Surajit Some, Hyeon Jun Hwang, Byoung Hun Lee

**Affiliations:** 1 Department of Electrical Engineering, Center for Semiconductor Technology Convergence, Pohang University of Science and Technology, Cheongam-Ro 77, Nam-gu, Pohang, Gyeongbuk 37673 Republic of Korea; 2 School of Materials Science and Engineering, Gwangju Institute of Science and Technology, Cheomdan-Gwagiro 123, Buk-gu, Gwangju, 61005 Republic of Korea; 3 Department of Specialty Chemicals Technology, Institute of Chemical Technology, Matunga, Mumbai, 400019 India

**Keywords:** Electrical and electronic engineering, Electronic devices

## Abstract

*P*-type ternary switch devices are crucial elements for the practical implementation of complementary ternary circuits. This report demonstrates a *p*-type ternary device showing three distinct electrical output states with controllable threshold voltage values using a dual-channel dinaphtho[2,3-*b*:2′,3′-*f*]thieno[3,2-*b*]-thiophene–graphene barristor structure. To obtain transfer characteristics with distinctively separated ternary states, novel structures called contact-resistive and contact-doping layers were developed. The feasibility of a complementary standard ternary inverter design around 1 V was demonstrated using the experimentally calibrated ternary device model.

## Introduction

Recently, multi-valued logic (MVL) technology has attracted interest as an alternative architecture to address the rapid increase in energy consumption required for massive data processing^1–5^. MVL provides significant efficiency gains in terms of the number of transistors and the interconnection length required to perform equivalent functions designed with binary logic. In particular, the ternary logic that is composed of three logic states—0, 1, and 2—has several advantages over Boolean logic design using the conventional complementary metal–oxide–semiconductor (CMOS) logic, with the possibility of the lowest power consumption among MVLs^6–9^.

Owing to the apparent merit of architectural simplicity, various kinds of ternary devices have been investigated for many decades. Among them, carbon nanotube field-effect transistors (CNTFETs) have been the most widely investigated, but all studies using CNTFETs have been theoretical because it is difficult to arrange various types of CNTs with different threshold voltages (*V*_th_) in the form of integrated circuits^7,8^. More recently, a quantum dot gate FET was also proposed for ternary logic operation, but the process of fabricating two layers of well-aligned quantum dots is difficult to control and further scalability can be a serious challenge because the size of the quantum dots and the separation distance between them cannot be modulated flexibly^9^. Ternary devices using transition metal dichalcogenide (TMDC) heterojunctions have also been proposed to utilize the negative differential resistance or negative differential transconductance^10–13^. Shim et al. reported a ternary device based on a BP/ReS_2_ heterojunction forming a broken-gap band structure^10^. Nourbakhsh et al. demonstrated a MoS_2_/WSe_2_ ternary device with band-to-band tunneling mechanism^11^. Although these devices show promising functional feasibility, large-scale device integration remains quite challenging for TMDC materials. The wafer scale growths of TMDCs above oxides such as crystalline SiO_2_ or sapphire have been recently researched, but the direct growth on the oxide when the device structure exists is not desirable because of high growth temperatures over 800 °C^14,15^.

Graphene FETs are also used for ternary logic applications because of their high mobility, linearly controllable Fermi levels, and large area growth via thermal chemical vapor deposition (TCVD)^16–18^. Several articles have reported on ternary graphene FETs (GFETs), where external doping processes have been used in graphene channels to customize various PN junctions in the channel region^19–21^. However, the low noise margins because of the low on/off current ratios of GFETs, which are less than ~ 10, constitute a serious drawback in ternary circuits.

Kim et al. demonstrated an *n*-type ternary graphene barristor by forming dual junctions with different *V*_*th*_^22^. The graphene barristor, composed of graphene–semiconductor heterojunction, is the Schottky barrier triode modulating by control of the Fermi level of graphene using an electrical gate field. For implementing a ternary logic device, a graphene barristor is a better option, because of its linear drivability of current and large on/off ratio than GFETs. However, the benefits of ternary logic could not be fully accomplished by the resistive-load approach; a *p*-type counterpart ternary device is necessary to overcome this limit. Unfortunately, there are not many candidate materials for the *p*-type semiconductor that can be deposited at low temperatures, without damaging the graphene channel at low temperatures. However, several methods of fabricating *p*-type “binary” graphene barristors have been reported using pentacene and dinaphtho[2,3-*b*:2′,3′-*f*]thieno[3,2-*b*]-thiophene (DNTT), despite the relatively low field-effect mobility ~ 2 cm^2^/Vs^23–26^. In addition, DNTT showed better air stability than pentacene^27,28^. Thus, it is worthwhile to pursue a *p*-type ternary graphene barristor utilizing the works on air-stable DNTT–graphene barristors.

In this study, we investigated a dual-channel *p*-type ternary graphene barristor using a DNTT–graphene heterojunction. Multi-*V*_th_ *p*-type DNTT–graphene barristors with dual-channel structures were successfully demonstrated by modulating the carrier transport through the DNTT and metal contact interface. Subsequently, the functionalities and benefits of a standard ternary inverter (STI) were examined using the complementary ternary graphene barristors (ZnO–graphene barristor for an *n*-type device and DNTT–graphene barristor for a *p*-type device).

## Methods

### Device fabrication process

**Figure 1:**
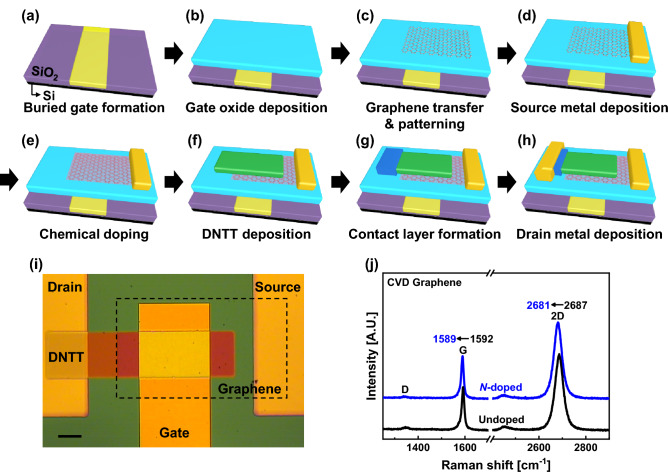
(**a**–**h**) Schematics of the fabrication process for a *p*-type graphene barristor with graphene chemical doping and a CRL/CDL. (**i**) Optical image of the fabricated device. (**j**) Raman spectra of CVD graphene with PEI concentrations of 0 and 0.01 wt% as undoped and *n*-doped, respectively.

Figure 1a–h show the fabrication process of a *p*-type graphene barristor with the DNTT–graphene heterojunction device. In this study, a buried gate structure was adopted to control the Fermi level of graphene more stably (Fig. 1a). Firstly, an oxide trench with 70 nm depth was formed on a 90 nm SiO_2_/Si substrate using photolithography and reactive ion etching with Ar and CF_4_ plasma. Subsequently, Au/Ti (60/10 nm) metals were deposited using an e-beam evaporator to fill the oxide trench. The buried gate pattern was formed in the trench region by applying a combination of lift-off and chemical–mechanical polishing processes. The buried gate structure was chosen because it can provide a more uniform electric field in the channel region than the bottom gate structure that is commonly employed in the early stages of graphene device studies^29^. This step was followed by atomic layer deposition of 30 nm of Al_2_O_3_ gate dielectric at 200 °C and annealed at 300 °C in a high vacuum (~ 10^−6^ Torr) for 1 h to improve the quality of the gate dielectric (Fig. 1b). Then, single-layer graphene grown by TCVD was transferred to the gate dielectric via a vacuum dry transfer process. The vacuum dry transfer process was used to maintain the electrical quality of graphene by minimizing the interfacial contamination from air molecules trapped at the interface between the graphene and oxide^30^. After the graphene transfer, a 30 nm Au hard mask layer was deposited to prevent the adverse influences of residual photoresist originating from successive photolithography. The graphene channel pattern with a metal hard mask was formed using photolithography and metal wet etching. Then, the graphene channel region was patterned with O_2_ plasma etching (Fig. 1c). Consequently, the channel region (480 × 520 μm) was initially protected by the Au hard mask, which was later removed to expose the graphene channel region before the source contact formation process. After the channel patterning, a 50 nm Au source electrode was formed on one side of the graphene channel using a shadow mask (Fig. 1d).

Following the source electrode formation, graphene doping was performed (Fig. 1e). The graphene channels were immersed in ethanol-diluted 0.0025–0.01 wt% polyethylenimine (PEI) Sigma Aldrich solution for 3 h and then briefly rinsed with a pure ethanol solution to prevent excessive doping.

After carefully preparing the graphene channel region, 50 nm of DNTT (Sigma Aldrich), which is a *p*-type organic semiconductor material, was thermally evaporated on the graphene channel to form a DNTT–graphene Schottky junction using a shadow mask (Fig. 1f). Upon completion of the DNTT deposition, 7 nm of Al_2_O_3_ was deposited by atomic layer deposition at 100 °C as a contact-resistive layer (CRL) for certain devices, whereas for other devices, a contact-doping layer (CDL) was formed with 10 nm of a 7:1 co-evaporation layer of DNTT and 2,3,5,6-tetrafluoro-7,7,8,8-tetracyanoquinodimethane (F_4_TCNQ, Sigma Aldrich) deposited on the drain region using a shadow mask (Fig. 1g). Finally, 50 nm of thermally evaporated Au drain electrode was formed using a shadow mask (Fig. 1h). An optical image of the final device structure is shown in Fig. 1i. The dimensions of the DNTT–graphene Schottky junction, which was directly controlled by the buried gate, were 200 × 300 μm.

### Evaluation of graphene doping by Raman spectroscopy

During device fabrication, the quality of the graphene channel was examined using Raman spectroscopy, as shown in Fig. 1j. The 2D/G area ratios for the undoped and 0.01 wt% *n*-doped graphene channels were 5.1 and 5.3, respectively, which confirmed that the graphene channel was a monolayer. Subsequently, the undoped graphene channel was slightly *p*-doped with impurity charges at the graphene–Al_2_O_3_ interface and the redshifts of the G and 2D peaks indicated that the PEI doping process successfully changed the doping state of the graphene channel to *n*-type^31^.

### Electrical characterization of the fabricated devices

The electrical properties of the fabricated devices were characterized using a semiconductor parameter analyzer (Keithley 4200) at room temperature.

## Results and discussion

### Operation of DNTT–graphene barristor and its ***V***_th_ modulation using graphene chemical doping

**Figure 2:**
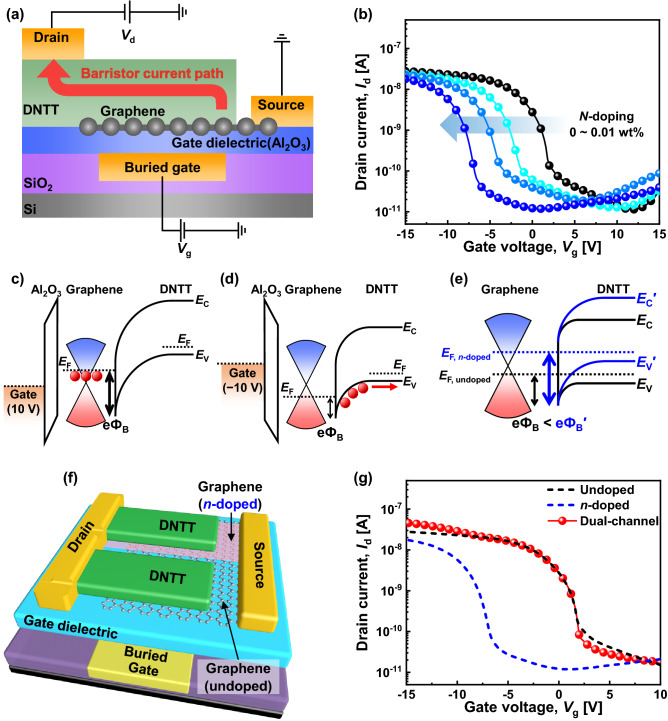
(**a**) Cross-sectional structure of graphene barristor with the direction of current flow. (**b**) Transfer curves for the *p*-type graphene barristor with PEI concentrations of 0 (no doping), 0.0025, 0.005, and 0.01 wt% at *V*_d_ =  − 2 V. Schematic band diagrams of graphene barristor at (**c**) *V*_g_ = 10 V and (**d**) *V*_g_ =  − 10 V. (**e**) SBH difference between undoped and *n*-doped graphene barristors. (**f**) Schematic device structure of dual-channel *p*-type graphene barristor. (**g**) Transfer curves for single- and dual-channel devices at *V*_d_ =  − 2 V.

Figure 2a shows a schematic cross-sectional diagram of a *p*-type graphene barristor, where the arrow indicates the direction of carrier flow. The DNTT partially overlaps the graphene channel, and the Schottky junction is formed at the DNTT–graphene interface. The flux of charge carriers through the DNTT–graphene Schottky barrier can be modulated using the buried gate bias (*V*_g_), which modulates the Fermi level of graphene and thereby adjusts the Schottky barrier height (SBH).

The transfer curves of *p*-type graphene barristors are shown in Fig. 2b. When *V*_*g*_ is low (more positive *V*_g_), the graphene barristor is turned off because the SBH is too high to allow the flow of hole carriers, as shown in Fig. 2c. As *V*_g_ increases (more negative *V*_g_), the graphene barristor is turned on as the SBH is reduced, as illustrated in Fig. 2d. Because the current flow of the DNTT–graphene barristor is modulated by the SBH, the initial barrier height should be adjusted appropriately to achieve the desired *V*_th_. Therefore, the surface of the graphene channel region was treated with PEI solutions of various concentrations, viz., 0 (no doping), 0.0025, 0.005, and 0.01 wt%. As the concentration of the PEI solution increases, *V*_th_ of the graphene barristor shifts toward negative *V*_g_; *V*_th_ is − 6.8 V in the case of 0.01 wt% PEI concentration. The *n*-type dopant, PEI, induces additional electron charges in graphene, which shifts the Fermi level of graphene upward and increases the SBH. The effects of graphene doping are schematically illustrated by comparing the band diagrams of undoped and *n*-doped graphene barristors in Fig. 2e. To confirm the doping results, the SBHs were extracted from the output curves of the graphene barristor. For *V*_g_ ranging from − 15 to 15 V, the extracted SBHs are modulated from 0.19 to 0.34 V for the undoped device and from 0.21 to 0.39 V for the *n*-doped device, indicating that the *V*_th_ shift of the graphene devices is caused by the change in the SBH due to chemical doping. (The SBH extraction method is described in Supplementary information, Fig. S2).

In principle, it is possible to obtain a switching device with three different output current levels by connecting two graphene channels doped with different doping concentrations, i.e., different *V*_th_, in parallel. Figure 2f shows a schematic diagram of a dual-channel graphene barristor having two channels with different *V*_th_ connected in parallel. One side is undoped, whereas the other side is 0.01 wt% *n*-doped graphene. The transfer curve of the dual-channel graphene barristor is shown as red solid circles in Fig. 2g. The intention of this device design is that the undoped channel is turned on first and saturated to obtain an intermediate-current state and the *n*-doped channel operates later to form an on-current state. However, Fig. 2g shows that it is not easy to distinguish between the intermediate-current and on-current states of a dual-channel graphene barristor, because the current level of the *n*-doped channel is lower than that of the undoped channel. Thus, only a slight increase in the high *V*_g_ region is observed. Therefore, the current levels and shapes of the transfer curves of the two-channel regions should be carefully optimized to achieve stepwise ternary transfer characteristics. The methods and strategies used to modulate the transfer characteristics of two graphene channels to obtain distinctly separated ternary transfer characteristics were as follows.

### Current engineering of DNTT–graphene barristor with contact resistance modulation

**Figure 3:**
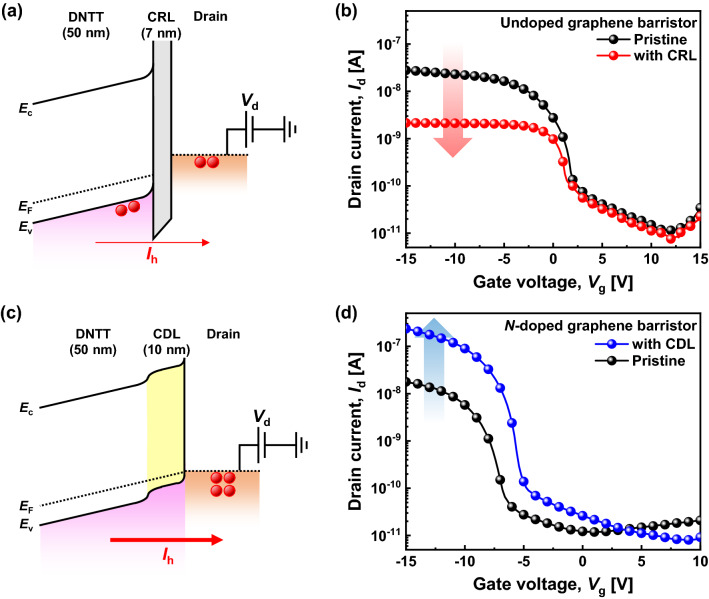
(**a**) Schematic band diagram with CRL. (**b**) Transfer curves of undoped graphene barristors with and without CRL at *V*_d_ =  − 2 V. (**c**) Schematic band diagram with CDL. (**d**) Transfer curves of *n*-doped graphene barristors with and without CDL at *V*_d_ =  − 2 V.

First, the saturation characteristics of the graphene channel with low *V*_th_ should be improved to obtain a more flat saturation curve, because a flat intermediate-state level is desirable to improve the noise margins of ternary logic circuits. Therefore, we introduced a thin Al_2_O_3_ layer between the DNTT and drain contact metal to add a series resistance component. The schematic band diagram of a graphene barristor with a thin Al_2_O_3_ layer is shown in Fig. 3a. After inserting the thin Al_2_O_3_ layer, the drain current is slightly degraded, but the saturation portion of the *I*–*V* curve becomes more flat, as shown in Fig. 3b. We named this layer a CRL. Because the CRL does not interfere with the Schottky junction between the graphene and DNTT, *V*_th_ of the device is minimally affected.

Second, it was required to improve the on-current level of the graphene channel with high *V*_th_ to obtain a more distinct level 2. In this case, the carrier concentration of the DNTT needed to be higher to reduce the contact resistance, and hence, F_4_TCNQ, which is a *p*-type dopant for DNTT, was employed. F_4_TCNQ improves the conductivity of DNTT by creating an additional hole carrier in the DNTT region. F_4_TCNQ was thermally co-evaporated with DNTT at a 7:1 ratio. This 10-nm-thick F_4_TCNQ layer, which is called a CDL, effectively lowers the hole injection barrier between Au and DNTT and enhances the current flow through the DNTT^32–34^. A schematic band diagram of the graphene barristor with the CDL is provided in Fig. 3c. The transfer curve of the graphene barristor exhibits an increase of nearly one order of magnitude in the drain current after adding the CDL, as shown in Fig. 3d, while simultaneously shifting *V*_th_ slightly to the positive side.

The influences of CRL and CDL on the contact resistance were experimentally measured using the transfer length method (Supplementary information, Fig. S3). The contact resistances of the DNTT/drain electrode contact with CRL and CDL were 16.2 and 0.1 MΩ cm, respectively, whereas the initial contact resistance was 0.7 MΩ cm.

By strategically combining the effects of the PEI doping of graphene and the insertion of a CDL or CRL before the metal contact formation, it became possible to shift and reshape the transfer curves of dual-channel ternary graphene barristors and to obtain satisfactory ternary *I*–*V* characteristics.

### Demonstration of *p*-type ternary barristor with a saturated intermediate current state

**Figure 4:**
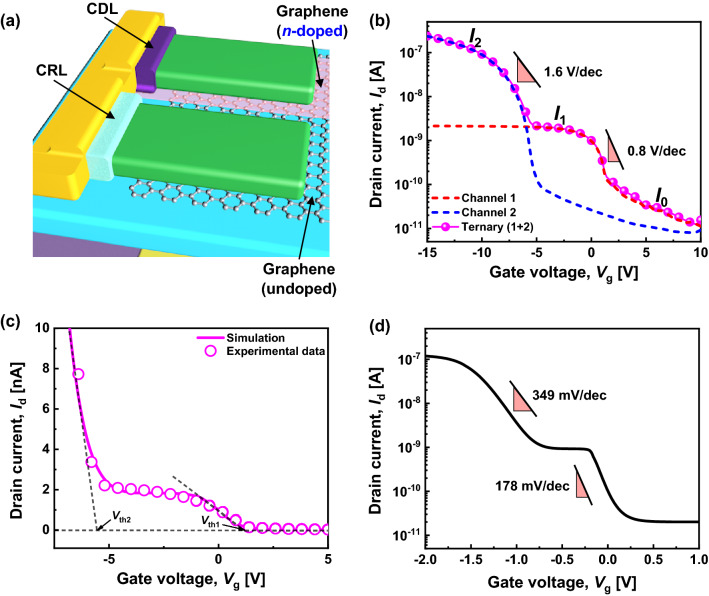
(**a**) Schematic device structure of dual-channel *p*-type ternary graphene barristor with a CRL and CDL. (**b**) Transfer curves for each channel and the ternary device at *V*_d_ =  − 2 V. *V*_th_ of the ternary device is defined as the cross points of the off-current level and the transition current tangent. (**c**) Transfer curve of the ternary device on a linear scale and its fitting result. (**d**) Transfer curve of a *p*-type ternary device modelled with EOT = 1 nm and *V*_d_ =  − 1 V.

For ternary logic operation, two channels were placed in parallel; one was the undoped graphene barristor channel with a CRL, which provided a saturated intermediate state (channel 1), and the other was the *n*-doped graphene barristor channel with a CDL, which provided the on-state (channel 2), as shown in Fig. 4a. The resulting transfer curve of the *p*-type ternary device is presented in Fig. 4b. In comparison with Fig. 2g, the separation of the current with three states, viz., 0 (off-state), 1 (intermediate state), and 2 (on-state), is much more pronounced. Furthermore, the current ratios between states are balanced as 10^2^, which is sufficient to divide the current level between ternary states. This is the first demonstration of a *p*-type ternary logic switch with a saturated intermediate state. Figure 4c shows *I*_d_–*V*_g_ of the *p*-type ternary device on a linear scale. The saturation of the intermediate current is distinctly observed even on this scale, which is important for the ternary logic gate circuit. Another positive result of this study is that the electrical characteristics of the *p*-type ternary device can be maintained for more than 45 days, confirming that this device is air-stable (Supplementary information, Fig. S4).

The performance of the ternary device is not yet competitive with CMOS technology on a similar scale. The operation voltage range is quite large, and the subthreshold swings remain very high due to the thickness of the gate dielectric (30 nm of Al_2_O_3_). In principle, further performance enhancement is quite feasible because there are several structural parameters, such as the electrical thickness of the gate dielectric, thickness and doping state of the DNTT, and area of the Schottky junction, that can be optimized further. Because the experimental optimization requires a rather extensive material-based study, theoretical analysis of the future scalability and performance was performed based on the fitting result shown in Fig. 4c to assess the competitiveness of this device for practical applications, especially with the objective of achieving 1 V operation.

### Ideal device model and complementary STI simulation

The electrical operation of the *p*-type ternary device was well-fitted by a capacitor-based ternary device model (Supplementary information, Fig. S5), which is commonly used to describe graphene barristors. Using this model, electrical characteristics with a satisfactory flat intermediate-state current were obtained, as shown in Fig. 4d. After several iterations of device modeling, a set of device parameters was determined, as summarized in Table 1. At an equivalent oxide thickness (EOT) of 1 nm, the subthreshold swings could be improved to 178 and 349 mV/dec for the first and second transitions, respectively.

**Table 1:** Device parameters for ternary device modeling, where A* is the Richardson constant and *N*_A_ and *N*_D_ are the charge concentrations of the semiconductors.

Parameters	EOT [nm]	*V*_dd_ [V]	*T* [K]	*W* [μm]	*L* [μm]	*A* [cm^2^]	A* [A/cm^2^K^2^]	*N*_A_, *N*_D_ [cm^−3^]	*R*_C_ *W* [MΩ cm]		
									Intermediate state	On-state	
*p*-type	1	1	300	200	300	6 × 10^−4^	120	1 × 10^16^	22 × 10^1^		1.6 × 10^−1^
*n*-type				30	15	4.5 × 10^−6^	34	1 × 10^18^	3.3		2.4 × 10^−3^

Then, a complementary STI was designed using the device model developed for 1 V operation, as shown in Fig. 4. For the *n*-type dual-channel ternary graphene barristor, the ZnO–graphene barristor model reported in the literature^22^ was used (Supplementary information, Fig. S6).

**Figure 5:**
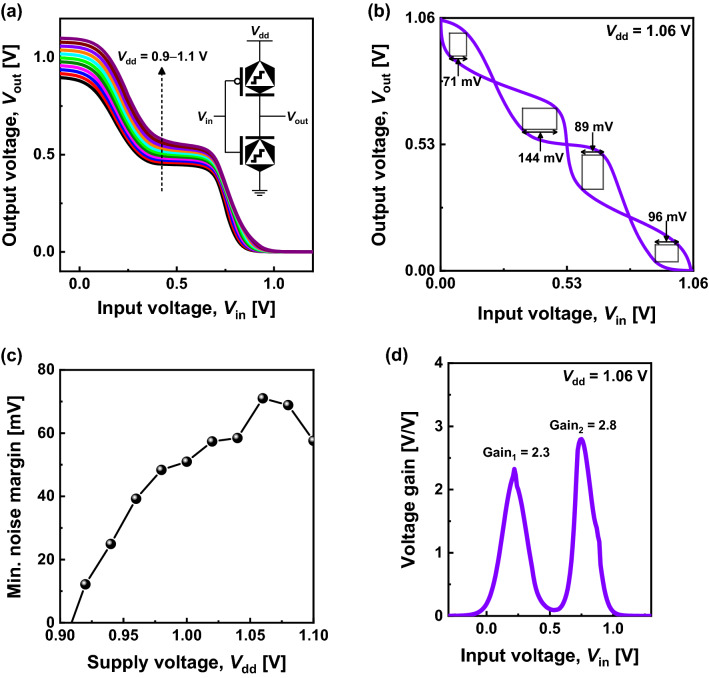
(**a**) Voltage transfer characteristics of STIs for *V*_dd_ = 1 ± 0.1 V (inset: schematic of the transfer level of a complimentary STI circuit). (**b**) Butterfly curve with noise margins of STI for *V*_dd_ = 1.06 V and (**c**) minimum noise margins extracted as a function of *V*_dd_. (**d**) Voltage gains of two-state transitions for *V*_dd_ = 1.06 V.

Figure 5a presents the voltage transfer characteristics of STI simulated with *SPICE* for *V*_dd_ = 1 ± 0.1 V. Well-behaving STI transfer characteristics were obtained around *V*_dd_ = 1 V. For STI, four noise margins were defined, as demonstrated in Fig. 5b. Figure 5c depicts the minimum noise margin as a function of V_dd_, in which the minimum noise margin was 71 mV at *V*_dd_ = 1.06 V. The very narrow peak region in Fig. 5c indicates that the operation margin of ternary STI is small and that well-balanced *n*-type and *p*-type ternary switches are necessary. The gains of the two-state transitions were 2.3 and 2.8, respectively, for *V*_dd_ = 1.06 V (Fig. 5d). As a result, a complementary STI was successfully demonstrated using only two ternary devices. It implies that more complex ternary logic circuits can be designed using fewer devices and shorter interconnect lengths than conventional Boolean logic circuits^6,7^. However, the large static power dissipation in the intermediate state due to the half turn-on of both *n*- and *p*-type ternary devices remains an issue for improvement for low-power systems (Supplementary information, Fig. S8).

## Conclusions

A fully functional *p*-type ternary device is the last essential element for the operation of complementary ternary logic circuits. By combining the demonstration of this device with the recent progress related to *n*-type ternary devices, reasonable projections of the performance and functionality of a complementary ternary inverter were achieved, and the feasibility of low *V*_dd_ operation at 1 V was confirmed. Although further optimization to scale down the device is necessary, this report confirms the achievement of a major milestone in ternary logic technology for extremely low-power computing.

## Supplementary Information

Supplementary Information.

## Data Availability

All data related to this paper can be requested from the corresponding author upon reasonable request.
