# Supplementary material for: Dual-channel P-type ternary DNTT–graphene barristor
Source: Sci Rep. 2022 Nov 12;12:19423. doi: 10.1038/s41598-022-23669-w (PMC9653424; doi:10.1038/s41598-022-23669-w)
Supplement: Supplementary file 1 — Supplementary Information. [file 41598_2022_23669_MOESM1_ESM.docx]

Supplementary Information

**Dual-Channel *P*-Type Ternary DNTT–Graphene Barristor**

Yongsu Lee^1^, Seung-Mo Kim^1^, Kiyung Kim^1^, So-Young Kim^1^, Ho-In Lee^1^, Heejin Kwon^1^, Hae-Won Lee^1^, Chaeeun Kim^2^, Surajit Some^1,3^, Hyeon Jun Hwang^1*^, Byoung Hun Lee^1*^

^1^Center for Semiconductor Technology Convergence, Department of Electrical Engineering, Pohang University of Science and Technology, Cheongam-ro 77, Nam-gu, Pohang, Gyeongbuk 37673, Republic of Korea

^2^School of Materials Science and Engineering, Gwangju Institute of Science and Technology, Cheomdan-gwagiro 123, Buk-gu, Gwangju 61005, Republic of Korea

^3^Department of Specialty Chemicals Technology, Institute of Chemical Technology, Matunga, Mumbai 400019, India

^*^Corresponding author. Email: [hhjune@postech.ac.kr](mailto:hhjune@postech.ac.kr), [bhlee1@postech.ac.kr](mailto:bhlee1@postech.ac.kr)

**Raman spectroscopy of DNTT**

Fig. S1 is the Raman spectroscopy of DNTT, which identifies itself.^S1^

**Fig. S1** Raman spectroscopy of DNTT.

**The extraction method of the Schottky barrier height (SBH) of the *p*-type DNTT–graphene barristor**

Fig. S2(a) shows output curves of *p*-type DNTT–graphene barristors according to various *V*_g_. For *V*_d_ > 0 V, graphene barristors allow on-current in all *V*_g_ ranges as the forward region of the Schottky diode. In the reverse region (*V*_d_ < 0 V), the drain current is more modulated by *V*_g_. For this reason, the reverse drain region is focused on device performance demonstration due to reverse regions showing a higher on/off current ratio than the forward drain regions.

The Schottky barrier heights ($\Phi_{B}$) of *p*-type graphene barristor with various *V*_g_ are extracted from the Schottky diode equation using drain current ($I$) vs. drain voltage ($V$) plot.^S2^

$I=I_{s}\left[ exp\left( \frac{qV}{\eta kT} \right)-1 \right], I_{s}=AA^{*}T^{2}exp\left( \frac{-q\Phi_{B}}{kT} \right)$ (S1)

$\ln\left[ \frac{I}{1-\exp\left( \frac{-qV}{kT} \right)} \right]=\frac{q}{\eta kT}V+\ln I_{s}$ (S2)

$\Phi_{B}=-\frac{kT}{q}\ln\left( \frac{AA^{*}T^{2}}{I_{s}} \right)$ (S3)

Where $q$ is the electron charge, $\eta$ is the ideality factor of the Schottky diode, $k$ is the Boltzmann's constant, $T$ is temperature, $A$ is the DNTT–graphene junction area, and $A^{*}$ is the Richardson constant of DNTT. $I_{s}$ is extracted from the equation (S2) in low forward $V$ regions, as shown in Fig. S2(b). Then $\Phi_{B}$ is calculated by the equation (S3) in a fixed $T$ as shown in Fig. S2(c). The SBH progressively adjusted by the doping are extracted as 0.34 eV, 0.25 eV, 0.19 eV for the undoped and 0.39 eV, 0.33 eV, 0.21 eV for the 0.01 wt% *n*-doped device at *V*_g_ = 15, 0, −15 V, respectively.

**Fig. S2** (a) Output curves of the undoped (left) and 0.01 wt% *n*-doped (right) graphene barristor according to *V*_g_ = −15, 0, and 15 V. (b) $I/\left[ 1-\exp\left( \frac{-qV}{kT} \right) \right]$ vs. $V$ plot example for undoped graphene barristor. (c) extracted $\Phi_{B}$ for undoped and 0.01 wt% *n*-doped graphene barristor.

**TLM measurement for extraction contact resistances**

The contact resistances between DNTT–Au electrodes of pristine, and with contact resistive layer (CRL) and contact-doping layer (CDL) were extracted by the Transmission line method (TLM) as shown in Fig. S3(a).

$R_{\mathrm{TLM}}=R_{SC}+{2R}_{C}$ (S4)

$R_{\mathrm{TLM}}$ is the total resistance of TLM, where $R_{\mathrm{SC}}$ is the resistance of a semiconductor, and $R_{C}$ is contact resistance between a semiconductor and a metal electrode. Here, the bulk resistance of metals is ignored as it is relatively small compared to others. $R_{\mathrm{SC}}$ is represented below equation:

$R_{\mathrm{SC}}=R_{S}\frac{L}{W}$ (S5)

Where $R_{S}$ is sheet resistance of the semiconductor, $L$, and $W$ are the length and width of the semiconductor channel, respectively. Therefore, $R_{\mathrm{TLM}}$ is

$R_{\mathrm{TLM}}=\frac{R_{S}}{W}L+2R_{C}$ (S6)

For normalized width resistance,

$R_{\mathrm{TLM}}W=R_{S}L+2R_{C}W$ (S7)

Following this equation, $R_{C}W$ can be extracted from the half of the y-intercept by the plot of $R_{TLM}W$ vs. $L$ as shown in Fig. S3(b). The extracted contact resistances are shown in Fig. S3(c).

**Fig. S3** (a) Optical image of TLM structure, in which CRL or CDL can be on top of the DNTT channel. Scale bar = 100 μm. (b) TLM measurement at *V*_g_ = 0 V, (c) extracted $R_{C}W$ for pristine, with CRL, and CDL.

**Air stability of the *p*-type DNTT–graphene ternary barristor device**

The ternary characteristics of the *p*-type ternary device over time were investigated as shown in Fig. S4. There was no significant difference in the transfer curves and the ternary characteristics were maintained for 45 days.

**Fig. S4** The transfer curves of the DNTT–graphene ternary barristor device after 0 and 45 days.

**Device model of graphene barristor with a charge conservation law and ideal devices at EOT = 1 nm**

The graphene barristor is following the Schottky diode equation as shown in equation (S1). Here, $\Phi_{B}$ is determined based on charge conservation for three capacitors contained in a graphene barristor which are oxide dielectric ($C_{\mathrm{ox}}$), semiconductor ($C_{\mathrm{sc}}$), and graphene quantum capacitors ($C_{\mathrm{graphene}}$), as shown in Fig. S5(a).^S3^ The charge conservation equation is following below:

$Q_{M}+Q_{\mathrm{grap}h\mathrm{ene}}+Q_{\mathrm{sc}}=0$ (S8)

Where $Q_{M}$ is the charge accumulated in the gate electrode, $Q_{\mathrm{grap}h\mathrm{ene}}$ is the charge on the graphene quantum capacitor, and $Q_{\mathrm{sc}}$ is the space charge on the semiconductor channel. And each charge is calculated as following equations:

$Q_{M}=\left( V_{gs}-V_{gr} \right){\epsilon_{ox}}/{t_{ox}}$ (S9)

$Q_{\mathrm{graphene}}=C_{q}V_{gr}=\frac{2q^{2}}{\hbar\nu_{f}\sqrt{\pi}}\left( \left| n_{G} \right| \right)^{1/2}V_{gr} , n_{G}=\left( \frac{qV_{gr}}{\hbar\nu_{f}\sqrt{\pi}} \right)^{2}$ (S10)

$Q_{\mathrm{sc}}=-\sqrt{2q\epsilon_{sc}N_{A}\emptyset_{sc}}$ (S11)

Where $V_{\mathrm{gr}}$ is the Fermi level shift of graphene from the Dirac point by external bias, $\epsilon_{ox}$ is the permittivity of the gate oxide, $t_{ox}$ is the thickness of the gate oxide, $q$ is the charge of an electron, $\nu_{f}$ is the Fermi velocity of the Dirac electron, $n_{G}$ is charge concentration of graphene, $\epsilon_{s}$ is the permittivity of the semiconductor, $N_{A}$ is the doping concentration of the semiconductor, and $\emptyset_{sc}$ is the surface potential of the semiconductor. Using these equations (S9–11), $V_{gr}$ is extracted in terms of $V_{gs}$, and then $\Phi_{B}$ is determined.

Fig. S5(b) shows the schematic diagram of a dual-channel ternary graphene barristor. Following this, the analytical modeling is implemented for the ternary device, which is well-fitted with experimental data as shown in Fig. S5(c).

Furthermore, Fig. S6 shows the device scaling and performance enhancement result for both *p*- (Fig. S6(a)) and *n*-type (Fig. S6(b)) ternary devices, using the device model and parameters in Table.1. The parameters of the *p*-type device are extracted from the experimental results, and those of the *n*-type device are referred from the literature.^20^

.

**Fig. S5** (a) The schematic diagram of capacitor–charge distribution for the device model of graphene barristor. (b) The schematic diagram of the device simulation model for the ternary device, and (c) the analytical fitting result in log scale.

**Fig. S6** The transfer curves of (a) *p*-type and (n) *n*-type ternary devices modeled with EOT = 1 nm for *V*_d_ = ∓ 1 V, respectively.

**Noise margins of STI for *V*_dd_**

Butterfly curves with noise margins of STI for *V*_dd_ = 0.9, 1, and 1.1 V are shown in Fig. S7. For *V*_dd_ = 0.9 V, two noise margins are undefined.

**Fig. S7** Butterfly curves with noise margins of STI for (a) *V*_dd_ = 0.9 V, (b) 1 V, and (c) 1.1 V.

**Static power consumption of the STI**

Static power consumption of the STI (Fig. 5(a)) *V*_dd_ = 1.06 V is shown in Fig. S8. Relatively static power consumption occurs in the intermediate logic state (~1 nW) because of the leakage current between *V*_dd_ to *V*_gnd_.

Ternary logic has advantages for low-power computing because the ternary logic systems can be built using fewer devices and shorter interconnects compared to binary logic systems. However, due to the intermediate state leakage current problem, static power consumption diminishes the merits of ternary logic.

The first approach to mitigate the issue is to reduce the level of intermediate state current so that static power consumption can be reduced. In this case, the performance can be degraded. Thus, this approach can only be used for extremely low-power applications.

The other approach is to have a different power rail for the middle state. This approach requires additional devices to define the middle state and an additional power rail for *V*_dd_/2. Four transistors configuration is the most popular approach for the single pole triple throw (SPTT) circuit scheme. At this point, this scheme is one of the most promising options for ternary logic applications.^S4^

**Fig. S8** Static power consumption of the proposed STI at *V*_dd_ = 1.06 V.

**References**

S1. Bhardwaj, B. S. *et al.* Raman Spectroscopic Studies of Dinaphthothienothiophene (DNTT). *Materials* **12**, 615 (2019).

S2. Di Bartolomeo, A. Graphene Schottky diodes: An experimental review of the rectifying graphene/semiconductor heterojunction. *Physics Reports* **606**, 1–58 (2016).

S3. Heo, S. et al. Very-Low-Temperature Integrated Complementary Graphene-Barristor-Based Inverter for Thin-Film Transistor Applications. *Annalen der Physik* **530**, 1800224 (2018).

S4. Lee, Y. *et al.* Demonstration of Anti-ambipolar Switch and Its Applications for Extremely Low Power Ternary Logic Circuits. *ACS Nano* **16**, 10994–11003 (2022).
